# A novel T→G splice site mutation of *CRYBA1/A3* associated with autosomal dominant nuclear cataracts in a Chinese family

**Published:** 2012-05-15

**Authors:** Zhenfei Yang, Dongmei Su, Qian Li, Fan Yang, Zicheng Ma, Siquan Zhu, Xu Ma

**Affiliations:** 1 Capital Medical University,Beijing Ophthalmology & Visual Sciences Key Lab, Beiing, China; 2 National Research Institute for Family Planning, Beijing, China; 3 WHO Collaborative Center for Research in Human Reproduction, Beijing, China

## Abstract

**Purpose:**

The purpose of this study was to identify the disease-causing mutation and the molecular phenotype that are responsible for the presence of an autosomal dominant congenital nuclear cataract disease in a Chinese family.

**Methods:**

The family history and clinical data were recorded. The patients were given a physical examination and their blood samples were collected for DNA extraction. Direct sequencing was used to detect the mutation. Transcription analysis of the mutant crystallin, beta A1 (*CRYBA1/A3*) gene was performed to verify whether the defective mutation had influenced the splice of the mature mRNA.

**Results:**

The phenotype of the congenital cataract in the family was identified as a nuclear cataract type, by using slit-lamp photography. Direct sequencing revealed a novel mutation IVS3+2 T→G in *CRYBA1/A3*. This mutation co-segregated with all affected individuals in the family, but was not found in unaffected family members nor in the 100 unrelated controls. Transcription analysis of the mutant *CRYBA1/A3* gene indicated that this mutation had influenced the splice of the mature mRNA.

**Conclusions:**

Our study identified a novel splice site mutation in *CRYBA1/A3*. This mutation was responsible for aberrant splicing of the mature mRNA and had caused the congenital nuclear cataracts in the family. This is the first report relating an IVS3+2 T→G mutation of *CRYBA1/A3* to congenital cataracts.

## Introduction

Congenital cataract is a leading cause of poor vision or blindness in children, which accounts for more than 1 million blind children in Asia and about 10% of the childhood blindness worldwide. Approximately 50% of all congenital cataract cases may have a genetic cause [1-3]. These cataracts are most frequently inherited as autosomal dominant traits, but can also be inherited in other forms of Mendelian inheritance. Congenital cataract is a clinically and genetically heterogeneous lens disorder [4]. According to morphology, the cataracts can be classified into several subtypes: whole lens, nuclear, lamellar, cortical, polar, sutural, pulverulent, cerulean, coralliform, and other minor subtypes [5]. Cataracts that are phenotypically identical can result from mutations at different genetic loci and can have different inheritance patterns. Conversely, cataracts with dissimilar phenotypes may result from mutations in a single gene or a gene family. To date, more than 40 genetic loci have been linked to congenital cataracts, and at least 26 genes have been cloned and sequenced, including crystallins, connexins, heat shock transcription factor-4, aquaporin-0, and the beaded filament structural protein-2 [6]. Among these candidate genes, crystallin genes and connexin genes represent a major proportion of the mutations identified in congenital cataract. These include αA-crystallin (*CRYAA*), αB-crystallin (*CRYAB*), βA1/A3-crystallin (*CRYBA1/A3*), βB1-crystallin (*CRYBB1*), βB2-crystallin (*CRYBB2*), γC-crystallin (*CRYGC*), γD-crystallin (*CRYGD*), γS-crystallin (*CRYGS*), connexins46 (*GJA3*), and connexins50 (*GJA8*) [7].

In this study, we applied a functional candidate approach to test the known crystallin and connexin genes in a Chinese family. A novel transversion mutation IVS3+2 T→G in *CRYBA1/A3*, which influences the splice of mature mRNA, was detected.

## Methods

### Clinical examination and isolation of genomic DNA

**Figure 1:**
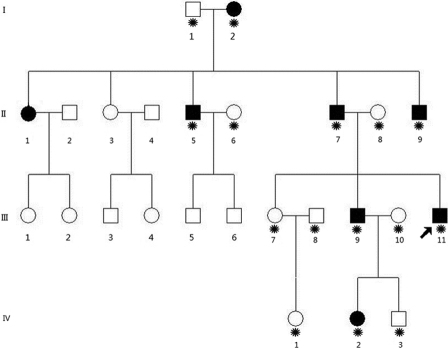
A Chinese family that has had autosomal dominant cataracts for four generations. The black symbols indicate individuals who have been given a diagnosis of congenital cataracts by doctors. The arrow indicates the proband. The asterisks indicate family members who participated in this study.

Data reaching back for four generations of a Chinese Han family with autosomal dominant congenital cataract was collected from Beijing Tongren Hospital (Figure 1). Information on 100 normal controls was also collected from the Tongren Hospital. The ethics committee of Capital Medical University approved the research and all participants from the family gave their informed consent. The study protocol followed the principles of the Declaration of Helsinki.

A history of cataract extraction or ophthalmologic examination was used to determine those whose status was considered to be affected, and all participating members also underwent ophthalmic examination, including visual acuity, slit-lamp examination, and intraocular pressure measurement. The phenotypes were documented by slit-lamp photography, and 5 ml of venous blood was collected in BD Vacutainers (BD, San Jose, CA) containing EDTA from participating family members and controls. Genomic DNA was extracted by QIAamp DNA Blood Mini Kits (Qiagen Science, Germantown, MD).

### Mutation detection

All coding exons and the flanking splicing junction of the candidate genes known to be associated with congenital cataract, including *CRYAA*, *CRYAB*, *CRYBA1/A3*, *CRYBB2*, *CRYGC*, *CRYGD*, *CRYGS*, *GJA3*, and *GJA8*, were amplified by PCR with primers listed in Table 1. Briefly, PCR amplification conditions were: Reaction Mixture Set Up (50 μl); 2 μl (40 ng/ml) of DNA, 1.5 μl (10 μM) of each exon primer, 20 μl of water , and 25 μl of PCR mix (2× EasyTaq PCR SuperMix; TransGen, Beijing, China) in a final reaction volume of 50 μl. Thermal cycling conditions were: an initial denaturation step at 95 °C for 5 min, 35 cycles (30 min at 95 °C, 30 min at 59°C, and 45min at 72 °C) and a final 10 min 72 °C extension. The PCR products were sequenced from both directions with the ABI 3730 automatic sequencer (PE Biosystems, Forster city, CA). The sequencing results were analyzed using Chromas 2.33 and compared to the reference sequences in the NCBI database.

**Table 1:** The primmers used for PCR.

**Exon**	**Forward (5′-3′)**	**Reverse (5′-3′)**
*CRYAA*-1	5′-AGCAGCCTTCTTCATGAGC-3′	5′-CAAGACCAGAGTCCATCG-3′
*CRYAA*-2	5′-GGCAGGTGACCGAAGCATC-3′	5′-GAAGGCATGGTGCAGGTG-3′
*CRYAA*-3	5′-GCAGCTTCTCTGGCATGG-3′	5′-GGGAAGCAAAGGAAGACAGA-3′
*CRYAB*-1	5‘-AACCCCTGACATCACCATTC-3′	5′-AAGGACTCTCCCGTCCTAGC-3′
*CRYAB*-2	5′-CCATCCCATTCCCTTACCTT-3′	5′-GCCTCCAAAGCTGATAGCAC-3′
*CRYAB*-3	5′-TCTCTCTGCCTCTTTCCTCA-3′	5′-CCTTGGAGCCCTCTAAATCA-3′
*CRYBA1*–1	5′-GGCAGAGGGAGAGCAGAGTG-3′	5′-CACTAGGCAGGAGAACTGGG-3′
*CRYBA1*–2	5′-AGTGAGCAGCAGAGCCAGAA-3′	5′-GGTCAGTCACTGCCTTATGG-3′
*CRYBA1*–3	5′-AAGCACAGAGTCAGACTGAAGT-3′	5′-CCCCTGTCTGAAGGGACCTG-3′
*CRYBA1*–4	5′-GTACAGCTCTACTGGGATTG-3′	5′-ACTGATGATAAATAGCATGAACG-3′
*CRYBA1*–5	5′-GAATGATAGCCATAGCACTAG-3′	5′-TACCGATACGTATGAAATCTGA-3′
*CRYBA1*–6	5′-CATCTCATACCATTGTGTTGAG-3′	5′-GCAAGGTCTCATGCTTGAGG-3′
*CRYBB2*–1	5′-GTTTGGGGCCAGAGGGGAGTGGT-3′	5′-TGGGCTGGGGAGGGACTTTCAGTA-3′
*CRYBB2*–2	5′-CCTTCAGCATCCTTTGGGTTCTCT-3′	5′-GCAGTTCTAAAAGCTTCATCAGTC-3′
*CRYBB2*–3	5′-GTAGCCAGGATTCTGCCATAGGAA-3′	5′-GTGCCCTCTGGAGCATTTCATAGT-3′
*CRYBB2*–4	5′-GGCCCCCTCACCCATACTCA-3′	5′-CTTCCCTCCTGCCTCAACCTAATC-3′
*CRYBB2*–5	5′-CTTACCCTTGGGAAGTGGCAATGG-3′	5′-TCAAAGACCCACAGCAGACAAGTT-3′
*CRYGC*-1	5′-TGCATAAAATCCCCTTACCG-3′	5′-CCTCCCTGTAACCCACATTG-3′
*CRYGC*-2	5′-TGGTTGGACAAATTCTGGAAG-3′	5′-CCCACCCCATTCACTTCTTA-3′
*CRYGD*-1	5′-CAGCAGCCCTCCTGCTAT-3′	5′-GGGTCCTGACTTGAGGATGT-3′
*CRYGD*-2	5′-GCTTTTCTTCTCTTTTTATTTCTGG-3′	5′-AAGAAAGACACAAGCAAATCAGT-3′
*CRYGS*-2	5′-GAAACCATCAATAGCGTCTAAATG-3′	5′-TGAAAAGCGGGTAGGCTAAA-3′
*CRYGS*-3	5′-AATTAAGCCACCCAGCTCCT-3′	5′-GGGAGTACACAGTCCCCAGA-3′
*CRYGS*-4	5′-GACCTGCTGGTGATTTCCAT-3′	5′-CACTGTGGCGAGCACTGTAT-3′
*GJA3*–1	5′-CGGTGTTCATGAGCATTTTC-3′	5′-CTCTTCAGCTGCTCCTCCTC-3′
*GJA3*–2	5′-GAGGAGGAGCAGCTGAAGAG-3′	5′-AGCGGTGTGCGCATAGTAG-3′
*GJA3*–3	5′-TCGGGTTCCCACCCTACTAT-3′	5′-TATCTGCTGGTGGGAAGTGC-3′
*GJA8*–1	5′-CCGCGTTAGCAAAAACAGAT-3′	5′-CCTCCATGCGGACGTAGT-3′
*GJA8*–2	5′-GCAGATCATCTTCGTCTCCA-3′	5′-GGCCACAGACAACATGAACA-3′
*GJA8*–3	5′-CCACGGAGAAAACCATCTTC-3′	5′-GAGCGTAGGAAGGCAGTGTC-3′
*GJA8*–4	5′-TCGAGGAGAAGATCAGCACA-3′	5′-GGCTGCTGGCTTTGCTTAG-3′

### Plasmid construction

DNA fragments extending from the terminal DNA fragment of intron 2 to the tail end of exon 4 of *CRYBA1* were amplified from the genomic DNA using the primer couples CRYBA1-F (5′-CGG GGT ACC ATA ACC ATC TAT GAT CAG GAG AAC-3′) and CRYBA1-R (5′-CCG GAA TTC CTC AAT GTG GTA GGC ATT ACT C-3′). PCR-amplified fragments carrying intron 3 and partial DNA fragments of exon 3 and exon 4 were 1,992 bp. Purified PCR products of *CRYBA1* were inserted into digested pcDNA3.1 vectors. The mutant construction was also created through PCR-mediated site-directed mutagenesis, with primers CRYBA1-M-F (5′-AGT GGC GCG GGA GTA TGG ACT TCC G-3′) and CRYBA1-M-R (5′-CCA TAC TCC CGC GCC ACT TTC CAC-3′). The wild-type and mutant plasmids were confirmed and named as pc3.1-CYRBA1-wild-type (wt) and pc3.1-CRYBA1 mutant-type (mt), respectively.

### Cell culture and transfection

The 293T cells were maintained in Iscove’s modified Dulbecco’s medium supplemented with 10% fetal bovine serum, 100 mg/ml penicillin and 100 mg/ml strep-tomycin, in a humidified atmosphere containing 5% CO_2_ at 37 °C. The expression vectors pc3.1-CYRBA1-wt and pc3.1-CYRBA1-mt were transfected into 293T cells using Lipofectamine 2000 (Life Technologies, Carlsbad, CA), according to lipofection procedures.

### RNA extraction and RT–PCR

The 293T cells were harvested 48 h after transfection. Total cellular RNA was extracted from the cells using the TRIzol Reagent (Life Technologies), according to the manufacturer’s protocol. An RNA PCR kit (TaKaRa, Dalian, China) was used to synthesize cDNA from 2 μg of RNA. After the RT reaction, the cDNA was amplified by using the two pairs of primers that were used in the PCR of the fragment DNA in *CRYBA1*. The amplified products were separated by electrophoresis on 2% agarose gels.

## Results

### Clinical evaluation

**Figure 2:**
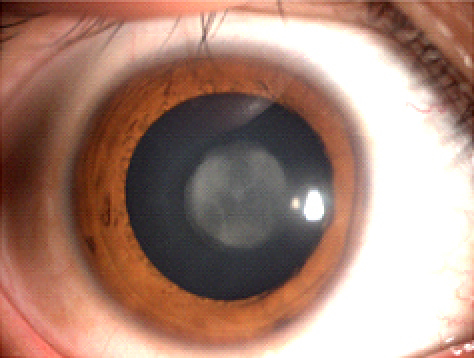
Slit-lamp photographs of the right eyes of probands. Slit-lamp examination revealed the opacity of the nuclear cataracts.

Slit-lamp examination revealed opacification of the nuclear cataracts (Figure 2).

### Mutation analysis

**Figure 3:**
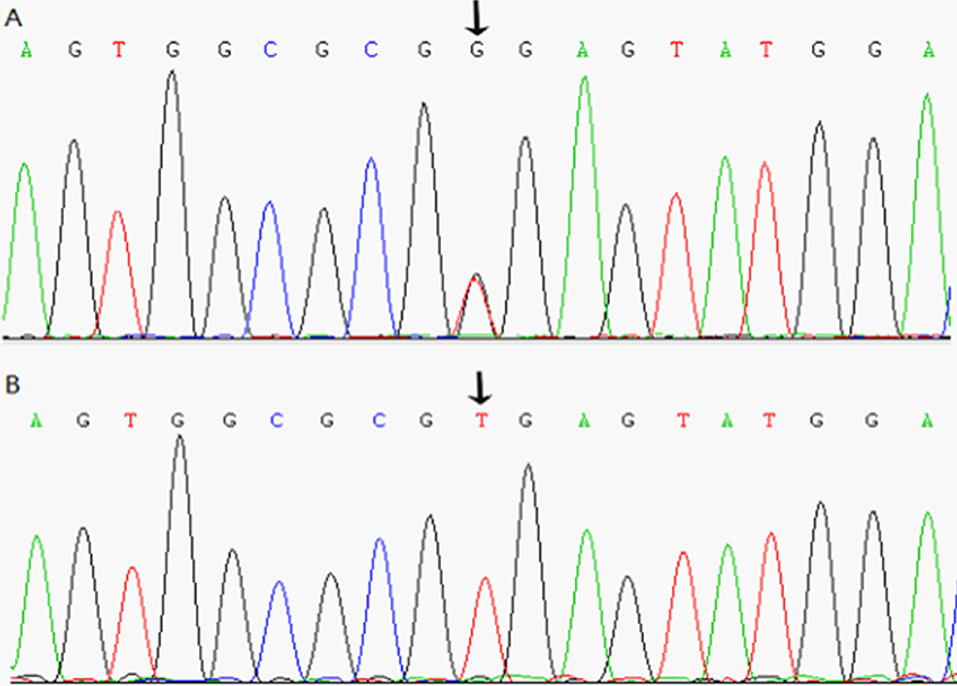
Partial sequence of *CRYBA1/A3* at exon3. **A**: Sequence of affected individual (individual III:11). **B**: Sequence of unaffected individual (individual III:7). In panel **A**, the heterozygous mutation IVS3+2 T→G was evident at the flanking splicing junction. This was identified in all the affected participants, but was not found in unaffected family members or in the 100 unrelated control subjects.

Through direct gene sequencing of the coding regions of the candidate genes, we identified an IVS3+2 T→G substitution in the donor splice site of intron 3 in *CRYBA1/A3* in all affected individuals (Figure 3). However, we did not find this mutation in any of the unaffected family members nor in the 100 unrelated controls. Indeed, we did not find any other mutations in this family, except for a few non-pathogenic single nucleotide polymorphisms (SNPs).

### Transcription analysis of the mutant *CRYBA1/A3* gene

**Figure 4:**
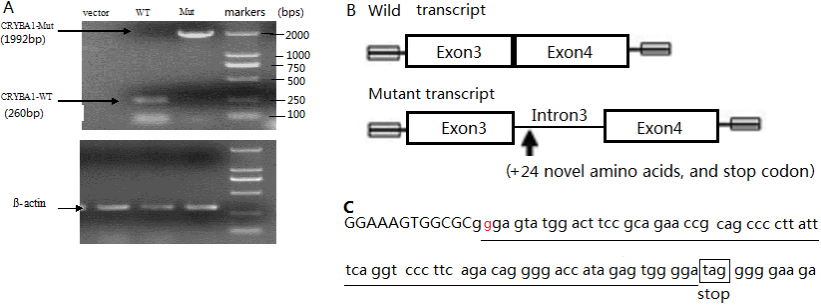
Transcription analysis of the mutant *CRYBA1/A3* gene. **A**: RT–PCR products separated on a 2% agarose gel. CRYBA1-wt (RT–PCR products from the 293T cells transfected with pc3.1-CYRBA1-wt) showed a minor band, about 260 bp; CRYBA1-mt (RT–PCR products from the 293Tells transfected with pc3.1-CYRBA1-mt) showed a major band, about 1,992 bp. **B**: Graphic presentation of wild and mutant transcript, with the wild type indicating that exon 3-exon 4 were combined, except for intron 3, and the mutant transcript indicating that exon 3-intron 3-exon 4 were combined. **C**: Flank sequence of splice mutation. The red font indicates mutant base, the additional 24 amino acid residues are marked by underline and the stop coden is marked by black frame.

The pc3.1-CYRBA1-wt and pc3.1-CYRBA1-mt expression vectors were transiently transfected into the 293T cells, to verify whether *CRYBA1* IVS3+2 T→G substitution influenced the splice of mature mRNAs. The RT–PCR products that were transfected from the 293T cells with pc3.1-CYRBA1-wt showed a minor band, about 260 bp, indicating that exon 3-exon 4 were combined, except for intron3. Meanwhile, those that were transfected from cells with pc3.1-CYRBA1-mt showed a major band, about 1,992 bp, indicating that exon 3-intron 3-exon 4 were combined (Figure 4A). The result indicated that IVS3+2T→G in *CRYBA1* led to incorrect splicing of *CRYBA1* mRNAs.

## Discussion

Lens crystallin is important in establishing and maintaining lens transparency [8]. Previous pedigree and transgenic animal research have indicated that mutation in crystallin genes would cause cataracts. Usually, the mutant crystallin has altered the stability, solubility, or ability to oligomerize and is predicted to precipitate from solution, which finally results in lens opacity. In addition to their roles in transmitting and focusing light, some lens crystallins have biochemical or enzymatic activity. For example, α-crystallin has a molecular chaperone function [9], and β-crystallin has been suggested as having a role in anoikis [10]. The βγ-crystallins share a highly stable structure comprising two domains joined by a connecting peptide. Each domain includes motifs that form a Greek key fold forming a β-sandwich structure. The γ-crystallins are found as monomers, while the β-crystallins associate into higher order complexes [11]. Most mutations described in the βγ-crystallins would be expected to cause major abnormalities in the protein structure, presumably resulting in an unstable protein that precipitates from solution and serves as a nidus for additional protein denaturation and precipitation, eventually resulting in the formation of cataracts.

*CRYBA3/A1*, located in 17q11.2, is a member of the β-crystallin family and consists of six exons encoding two proteins (βA3-crystallin and βA1-crystallin) from a single mRNA, through the use of an alternate translation initiation site. βA1/A3-crystallin consists of seven protein regions: four homologous (Greek key) motifs, a connecting peptide, and NH_2_- and COOH-terminal extensions. The NH_2_-terminal arm is encoded by the first two exons and the Greek key motifs are encoded by exon3–exon6 [12]. βA3-crystallin is identical to βA1-crystallin, except for 17 additional amino acid residues found on its NH_2_-terminal arm [13].

So far, in the *CRYBA1/A3* gene, three types of splicing mutations have been associated with autosomal dominant cataracts. And all three types were located in the first base in intron 3. The first one is the IVS3+1 G→A mutation [14-16]. The second type of mutation is the IVS3+1 G→C [17,18]. In 2011, we identified the third type, the IVS3+1 G→T mutation, in a Chinese family with suture cataracts [19]. This report, indicating the IVS3+2 T→G, will be the fourth type of splicing mutation as well as the first mutation found in the second base of intron 3 in *CRYBA1/A3*.

Several mutations at the absolutely conserved intronic 5′-gt or 3′-ag dinucleotide, at splice sites, have been identified in various genetic disorders. Splice-site mutations usually lead to aberrant pre-mRNA splicing, which results in exon skipping, activation of cryptic splice sites, creation of a pseudo-exon within an intron, or intron retention [20]. There has been little study of the mechanisms for normal pre-mRNA splicing in *CRYBA1/A3*. In this study, the T at position +2 of the 5′ (donor) splice site is highly conserved and mutation of this base was shown to disrupt the splice site, causing intron 3 to be retained during pre-mRNA splicing (Figure 4B). As a result of the mutation, the next 25 codons within the retained intron 3 would be a UAG stop site (Figure 4C), and the premature termination codon would cause truncated protein that contains the first motif and an additional peptide with 24 amino acid residues. According to the previous study, it is not possible to form even a single functional Greek key structure without the second motif. This is because the fourth strand that form the first motif is provided by the second motif, and vice versa [12].

Consequently, the protein would be folded incorrectly after synthesis. Not only would this improperly folded crystallin be unstable and serve as a nidus for precipitation of other damaged proteins, but it might also interfere with other appropriate associations of β-crystallin. Any of these events would be likely to result in the formation of cataracts. This suggests that the βA3-crystallin splice mutation might cause the cataracts in this family.
